# Eye Tracking in Virtual Reality: Vive Pro Eye Spatial Accuracy, Precision, and Calibration Reliability

**DOI:** 10.16910/jemr.15.3.3

**Published:** 2022-09-07

**Authors:** Immo Schuetz, Katja Fiehler

**Affiliations:** Justus Liebig University Giessen, Germany

**Keywords:** Eye Tracking, Head-Mounted Display, Accuracy, Precision, Virtual Reality

## Abstract

A growing number of virtual reality devices now include eye tracking technology, which can facilitate oculomotor and cognitive research in VR and enable use cases like foveated rendering. These applications require different tracking performance, often measured as spatial accuracy and precision. While manufacturers report data quality estimates for their devices, these typically represent ideal performance and may not reflect real-world data quality. Additionally, it is unclear how accuracy and precision change across sessions within the same participant or between devices, and how performance is influenced by vision correction. Here, we measured spatial accuracy and precision of the Vive Pro Eye built-in eye tracker across a range of 30 visual degrees horizontally and vertically. Participants completed ten measurement sessions over multiple days, allowing to evaluate calibration reliability. Accuracy and precision were highest for central gaze and decreased with greater eccentricity in both axes. Calibration was successful in all participants, including those wearing contacts or glasses, but glasses yielded significantly lower performance. We further found differences in accuracy (but not precision) between two Vive Pro Eye headsets, and estimated participants’ inter-pupillary distance. Our metrics suggest high calibration reliability and can serve as a baseline for expected eye tracking performance in VR experiments.

## Introduction

Eye tracking as a technique to estimate human gaze in relation to a variety of visual stimuli has proven highly successful over the years, whether as a research method in fields such as experimental psychology and cognitive science (29; 30; 57), as an assistive technology (34), or as a method for human-computer interaction (7; 15; 35). For a large part of its history, participants in eye tracking studies were usually seated, and their gaze was tracked relative to a two-dimensional monitor or screen. More recently, however, virtual reality (VR) technology has made great strides in quality and accessibility and subsequently found its way into many research labs, now making it possible to study dynamic human behavior in naturalistic but highly controlled virtual environments (8; 11; 20; 45; 57). With the advent of widespread and affordable consumer VR hardware, more and more head-mounted displays (HMDs) are now starting to include eye tracking technology out of the box. Besides the growing use in behavioral research, eye tracking in VR can enable a variety of different use cases (13, 14; 44). To highlight just some examples: Approaches such as foveated rendering allow higher visual fidelity and reduce rendering demands and power consumption for VR graphics (2; 43), gaze-based pointing and target selection can be utilized to create intuitive and multimodal methods of interaction (26; 35; 44; 55), and knowledge about a user’s current gaze direction can enable novel ways to experience and imperceptibly manipulate a virtual environment (e.g., 32; 37). All of these applications have different requirements relating to the quality of eye tracking data, such as high spatial accuracy and precision of the estimated gaze position in the case of gaze selection and interaction (17; 41; 50, 51), or very low latency between performing an eye movement and the corresponding change in a visual scene for foveated rendering (2; 54). Because the wide availability of eye tracking in VR is relatively recent, the current generation of consumer hardware often still falls behind research-grade devices in terms of data quality. Additionally, while accuracy and precision metrics are reported in the manual by most manufacturers, such values are typically best-case estimates and do not necessarily reflect the performance achievable under real-world conditions and with a diverse group of eye tracking participants (6; 16; 19; 39). Therefore, the main goal of the present study was a real-world evaluation of eye tracking performance for a specific VR HMD with built-in eye tracking, the HTC Vive Pro Eye (25).

A growing body of research highlights the importance of defining and reporting metrics of data quality in traditional screen-based eye tracking, starting with McConkie (38), who first suggested that researchers should describe the properties of the recorded eye tracking signal and any algorithms used in classification and analysis. More recently, Holmqvist, et al. (22) provided best-practice definitions for a variety of metrics such as spatial accuracy and precision. Generally, when measuring data quality, participants are instructed to fixate a target or set of targets with known position for a certain period of time while their gaze angle and/or position on the screen are recorded by the eye tracker. *Spatial accuracy* is then defined using the error or offset of the measured gaze relative to the target’s actual position, with larger error indicating lower accuracy. Accuracy may be reported as an angle of rotation in degrees (used for measurements of gaze angle, e.g. relative to a head-mounted eye tracker or VR HMD), as a distance in pixels or cm in the case of gaze position measurements on a screen plane, or as a distance in three-dimensional space if the intersection point of gaze direction and a virtual environment is used, such as when applying eye tracking in VR. Where accuracy measures the absolute deviation of the gaze estimate from a known target, *spatial precision* as defined by Holmqvist et al. (22) refers to the stability of individual measured gaze samples over time: Low precision indicates a wider spatial spread of the gaze samples belonging to a given target or fixation, while high precision implies that individual samples fall much closer to their average value. Multiple metrics for gaze precision have been proposed, with the two most common metrics being the Standard Deviation (SD) of gaze position or angle and the Root Mean Square error between individual samples (RMS). The achievable accuracy and precision in an eye tracking experiment can be significantly influenced by a variety of factors, such as participants’ eye physiology or vision correction (24; 39; 41) or the specific method employed to calibrate the eye tracking system (39). A recent review article by Holmqvist et al. (23) further summarizes factors that can influence eye tracking data quality and reiterates the need to define and report standardized descriptions and metrics when publishing eye tracking research.

Indeed, an extensive number of evaluations using a variety of metrics are now available for traditional screen-based eye tracking devices, which are likewise summarized in detail in the aforementioned review article. At the same time, the authors state that "little is known of the data quality of eye trackers integrated into VR goggles" (23). Because only a limited number of VR devices with built-in eye tracking have been released to date (see 54, for examples of current commercial models), relatively few systematic evaluations of the achievable gaze accuracy and precision specific to VR HMDs have been published so far. Lohr et al. (33) investigated the data quality of the SMI eye tracking add-on to the original HTC Vive, but this add-on is now discontinued after SMI was acquired by Apple in 2017. The authors report a mean accuracy of 0.67° and precision of 0.11° across saccade targets spanning ±15° horizontally and ±10° vertically (however, their mean absolute deviation measure of precision may not be directly comparable to the more widely used SD or RMS measures). The same HMD add-on was compared to a mobile eye tracker (SMI glasses) by Pastel et al. (42), who found comparable accuracy between the HMD and glasses in different fixation tasks (0.39°-0.51°), but reported RMS precision to be worse using the VR add-on (0.07° vs. 0.03°). Adhanom et al. (1) recently published an open-source package to measure gaze accuracy and precision within the Unity rendering engine. They report an average accuracy of 1.23° and RMS precision of 0.62° for 9 validation targets presented at a distance of 1 m in the HTC Vive Pro Eye HMD, but only show data for two participants. More recently, Sipatchin et al. (53) evaluated the same HMD for use in visual perimetry and tested 25 target positions spanning a range of ±26.6° in a head-fixed and head-free condition. They report an average accuracy of 4.16° and SD precision of 2.17° in their head-fixed condition, with accuracy decreasing noticeably at greater target eccentricities (around 8-10° at 26.6° eccentricity). Finally, a recent paper from our own group includes example data for a tutorial on VR behavioral studies and reports an average accuracy for the same HMD of around 0.5°, albeit for a limited field of view (FOV) of ±5° (N=5; 49).

Looking at the results summarized above, it is clear that the Vive Pro Eye headset is currently widely used for behavioral experiments in academic research labs. At the same time, the only data quality metric for this device that is available from the manufacturer is a spatial accuracy of 0.5° - 1.1° (25), with published accounts of measured eye tracking performance showing a large variation. To more closely determine the eye tracking performance that can be expected from this hardware during a real VR experiment, we here present a systematic evaluation of the spatial accuracy and precision achieved using the Vive Pro Eye HMD when using the Vizard VR rendering platform. We recorded data from eighteen participants, who repeatedly underwent the standard calibration procedure before performing a custom fixation task with 74 head-fixed target positions in the HMD. In contrast to the head-fixed task in Sipatchin et al. (53), who calibrated the eye tracker once at the start of the session and then presented multiple repetitions of each target, participants in our study performed ten separate measurement sessions over multiple days. This allowed us to quantify the reliability of the built-in calibration and describe participants’ individual accuracy and precision. Spatial metrics were collected over a span of ±15° horizontally and vertically using two separate Vive Pro Eye devices. This was done to assess potential variations in hardware performance: While devices are likely factory-calibrated and should not differ significantly in data quality, any such difference would be important to know for researchers planning a study and deciding whether their HMD needed to be individually characterized. Additionally, one of the two HMDs we tested (HMD 1, see below) was in active lab use for ca. 1.5 years while the other was new, allowing us to detect potential changes in metrics over the device life span. Besides potential hardware-related effects and beyond the metrics reported by Sipatchin et al. (53), we further investigated the influence of participants’ vision correction (glasses, contact lenses, or not wearing vision correction) on accuracy and precision across the visual field. Based on previous work (23; 39), we hypothesized that wearing vision correction should yield lower accuracy and precision than uncorrected vision.

In addition to the spatial performance of an eye tracking device, inter-pupillary distance (IPD) is a property of a participant’s individual eye and face geometry that is very important for VR. IPD generally refers to the horizontal distance between both eye pupils of an observer (9), and an accurate measure of the user’s IPD allows to correctly position the lenses within the HMD and adjust the virtual camera viewpoints from which the left and right eye images are rendered by the 3D engine. This ensures correct stereoscopic presentation and an immersive VR experience (48), and incorrect IPD settings in a VR HMD can even lead to visual discomfort (21). The eye tracker built into the Vive Pro Eye HMD reports the position of each pupil in a coordinate system referenced to the HMD itself, and this allows for a direct estimate of each participant’s IPD (in fact, one of the steps in the eye tracker’s automated calibration protocol is to help the wearer physically adjust the lenses to their IPD to achieve an optimal visual image). We therefore compare IPD values estimated using the eye tracker for each participant and session to those measured for distance viewing using an optometric pupilometer. If estimated values are accurate, this would facilitate better visual quality by setting correct viewpoint geometry and to skip optometric IPD testing as a separate step when testing participants in behavioral studies.

Our evaluation and metrics presented below can serve as a starting point when designing a study or interactive experience using the Vive Pro Eye eye tracker, and we give concrete recommendations on how to achieve optimal performance with this device based on our experience gained during data collection.

## Methods

### Participants

Eighteen volunteers (9 female, 9 male; mean age: 29 ± 7 years, range 20 to 49 years) took part in the experiment. Out of these, 6 persons wore glasses during the experiment, 6 wore contacts, and 6 wore no vision correction. Because we used two separate HMDs (see below), vision correction groups were balanced across both devices. All participants verbally reported good stereo vision and no history of oculomotor or neuromuscular deficits. In fourteen out of eighteen participants, we additionally measured stereo acuity using the Graded Circle Test (Stereo Optical, Chicago IL, USA) and their inter-pupillary distance (while fixating at optical infinity) using a pupilometer (Hangzhou Feng Hai Electronic Commerce Co. Ltd, Hangzhou, China). These additional measures were collected at a later date and four participants were no longer available for in-person testing due to having moved away at the end of the academic term. Stereo acuity was used to confirm verbal reports of good depth perception where available. Participants failing the first level of the Graded Circle Test (stereopsis of worse than 400 seconds of arc) would have been excluded from analysis, but all participants achieved stereopsis of 100 seconds of arc or better. Individual demographic, vision correction, and other relevant parameters are shown in supplementary Table S1. Participants were researchers or students in our lab, gave written informed consent and received no financial compensation for their participation. The experiment was approved by the research ethics board at Justus Liebig University Giessen, and was run in accordance with the Declaration of Helsinki (2008).

### Apparatus

Participants wore one of two Vive Pro Eye VR HMDs during the experiment. This headset has a display resolution of 1440 × 1600 pixels per eye, 90 Hz refresh rate and a FOV of 110° as stated by the manufacturer. Recent work by Sauer et al. (47) found the official FOV specifications for most VR headsets to be overstated and reports the effective FOV of the Vive Pro Eye at 94° horizontally. The HMD is equipped with an eye tracking system running at 120 Hz sampling rate and reported to achieve a spatial accuracy of 0.5° - 1.1° (25). For the purpose of the present study, we chose to sample eye position and orientation data once per display frame within the Vizard rendering loop (i.e., at 90 Hz). This is a common setup in VR eye tracking experiments, because it avoids the need for additional background data recording processes outside of the rendering engine. We selected this method to more closely measure typical rather than theoretical best performance in a VR experiment. To investigate potential differences in calibration and data quality between devices, two separate units were used, hereafter labeled *HMD 1* and *HMD 2*, which were pseudo-randomly assigned to participants while balancing vision correction groups. Single-use disposable paper covers were applied to the HMD before testing (VRCover, Gauss Labs Limited, Hong Kong), and participants and the experimenter wore surgical face masks due to the ongoing COVID-19 pandemic.

The experiment was implemented in Python using the Vizard VR toolkit (version 6.3; WorldViz, Santa Barbara, CA, USA), SRanipal software development kit (SDK; version 1.1.2.0), SteamVR (version 1.17.16) and our in-house software toolbox for behavioral experiments in VR (vexptoolbox version 0.1.1; 49). Each participant was tested using one out of three separate lab setups, subject to room availability. Two SteamVR 2.0 base stations ("lighthouses"; Valve Corp., Bellevue, WA, USA) were set up in each lab room for positional tracking, and each lab was equipped with a VR-capable desktop computer (Lab 1: Intel Core i9 CPU, 3.60GHz, 32 GB RAM, NVidia GeForce RTX 3080 GPU; Lab 2: Intel Core i9 CPU, 2.6 GHz, 32 GB RAM, Dual NVidia GeForce GTX1080 Ti GPU; Lab 3: Intel Xeon W2135 CPU, 3.7 GHz, 32 GB RAM, 2 GB NVidia Quadro P2200 GPU). Lab setups were used equally often, but could not be completely balanced across factors such as HMD and vision correction due to availability and the relatively low number of participants (for the lab setup used for each participant, see Table S1).

### Procedure

Each participant performed a total of ten *measurement sessions*. In each session, the participant was first seated in the lab and fitted with the HMD if necessary. To better approximate variability seen in real-world lab experiments, where participants might be invited for multiple sessions, they were encouraged to take off the HMD between sessions and perform the ten sessions over the course of multiple days, although this was not a requirement. Participants took between one and seven individual testing days to complete the experiment (cf. Table S1). At the start of each session, participants were shown a simple virtual environment consisting of a wooden tiled floor and floating instruction text. After a key press, the eye tracker’s built-in *calibration* provided by the SRanipal SDK was performed. In brief, this process guides the wearer through correctly fitting the HMD onto their head and adjusting the lenses for their individual inter-pupillary distance (IPD) using a knob on the HMD, then presents five calibration positions for the user to fixate in sequence ("follow the dot"). After all five positions were fixated, the calibration process ends and reports calibration success or failure.

After a successful calibration, participants pressed a key to start the main *validation* procedure. The SRanipal SDK only provides a calibration routine but no validation mechanism or numerical measure of accuracy, at least when running under Vizard. Therefore, validation was entirely performed by our Python code. During validation, target stimuli of known position were presented in sequence, and the participant was instructed to fixate the currently visible target. Validation targets consisted of a black sphere (radius: 0.1°) superimposed on a white disc (radius: 0.5°) and were presented at two different depths (distances from the participant’s eyes; near: 0.5 m and far: 6 m). Targets were always presented in a fixed position relative to the participant’s head position (head locked) and superimposed on a fronto-parallel background plane that extended beyond the horizontal and vertical FOV. To avoid measurement errors due to large variation in pupil size (12), the background plane was presented at a constant, medium gray color, keeping illumination within the HMD consistent. In order to sample spatial data quality across a large part of the field of view, target positions in the far (6 m) depth plane spanned ±15° and targets in the near depth plane (0.5 m) were shown at ±10° from straight ahead, yielding a total number of 49 + 25 = 74 trials per session. Each target was presented for a duration of 2 s and then changed color to green for another 0.2 s before disappearing. Participants were instructed to fixate the central black sphere of each target until the color change, and to blink after the color change if necessary. The next target was then presented automatically after an interval of 1 s. While Nyström et al. (39) reported that allowing the participant to confirm target fixation can yield higher calibration accuracy, this was not implemented here to standardize target presentation durations across all participants and sessions. Each measurement session lasted around 6 minutes and participants took around one hour in total to complete all measurement sessions.

**Figure 1.**
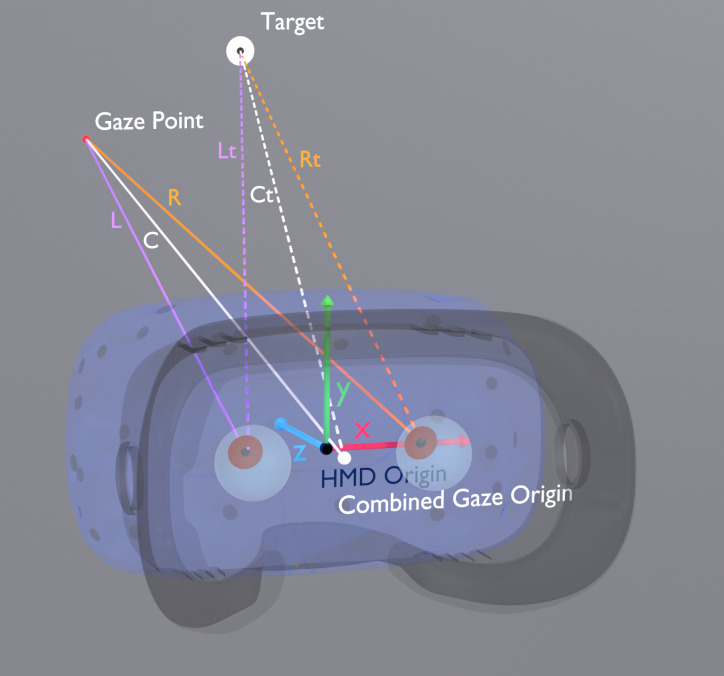
Depiction of the HMD with eye tracker coordinate system and relevant vectors. Small arrows represent the eye tracker’s coordinate frame, with its origin centered between the HMD’s lenses and x (red) towards the right eye, y (green) up and z (blue) representing the forward facing direction. Gaze direction vectors (solid lines) and gaze-target vectors (dashed lines) are shown for the left (L; purple), and right (R; orange) monocular gaze representations and the combined binocular gaze representation (C; white). Gaze error as defined in the text represents the angular offset between each solid and the corresponding dashed line (e.g. the angle between C and C_t_ represents gaze error for the combined gaze representation). HMD model based on “HTC Vive Pro” by user “Eternal Realm” on sketchfab.com (licensed CC-BY).

During each trial (presentation of a single target stimulus), we continuously recorded the *gaze origin* (generally equal to the position of the pupil within the HMD) and *gaze direction vector* for the left eye, right eye, and combined gaze representation in the HMD’s intrinsic frame of reference. Figure 1 gives an overview of the coordinate system and vectors involved. Pupil positions are specified relative to the center point between the HMD’s lenses (HMD origin), and gaze vectors are provided relative to a left-handed coordinate system with the positive X-axis pointing towards the right eye and the positive Z-axis pointing forward. Additionally, we recorded the vector from each gaze origin to the current target (*eye-target vector;* R_t_, L_t_, and C_t_ in Figure 1) as well as the headset’s position and orientation within the SteamVR (world space) coordinate system. Note that our code does not actually compute the combined gaze vector – both monocular and combined gaze representations are directly provided by Vizard and the SRanipal SDK and used as-is.

### Data Processing

Data were processed and analyzed using Python (version 3.8). Statistical analyses were performed in jamovi (version 2.2.5) and R (version 4.0.3). For each recorded trial, we computed *gaze error* in each sample as the angular difference between the gaze direction and eye-target vectors. Gaze error for left, right, and combined ("cyclopean") gaze was computed both as a combined error (absolute angular difference) and as individual horizontal and vertical errors. Note that in this manuscript, we use "horizontal" and "vertical" gaze error to refer to a horizontal or vertical deflection from the HMD’s forward direction (i.e., a yaw or pitch rotation, respectively), not to a rotation *around* the horizontal or vertical axis. The first 0.5 s (45 samples) of each trial were skipped to account for saccade latency and corrective saccades (4; 31; 50). We then selected the next 1 s of gaze data (90 samples) for further processing. The entire 90 samples were used for summary statistics and no fixation detection algorithm was used to refine data selection. This was done because event detection algorithms can produce very different results for the same dataset depending on the chosen parameters (3; 22; 28; 40), and we were interested in characterizing the performance of the eye tracking hardware without restricting the results to a specific combination of algorithm and parameters.

Spatial data quality metrics were defined as previously suggested by Holmqvist et al. (22). Because we used head-fixed targets and measured gaze angles relative to the headset’s frame of reference, we report all metrics in degrees. For the purpose of this manuscript, *accuracy* thus refers to the mean angular error between the eye-target and gaze directions, with lower errors indicating higher accuracy of the eye tracker’s gaze direction estimate. *Precision* was defined using the standard deviation (SD) of gaze error values as well as the inter-sample root mean square error (RMS).

In addition to accuracy and precision, prior work has shown that data loss or *invalid samples* can have an impact on data quality measures, such as when the pupil cannot be reliably detected by the eye tracker’s camera (22; 39). For the present study, eye tracking data collection was implemented using the Vizard sensor object functionality, which is the recommended way to access external hardware in Vizard due to its consistent interface. Unfortunately, this programming interface does not directly report a measure of data validity or confidence. Instead, we observed during piloting that when tracking of a sensor object is lost, such as when occluding a controller from the external base stations, Vizard repeats the most recent valid data sample until tracking is reestablished and new sensor data is available. Receiving multiple, exactly identical samples in sequence would otherwise be highly unlikely due to the inherent variability in the gaze estimation process. We therefore chose to report *repeated samples* as an indirect measure of sample validity, defined as the fraction of gaze error values in each trial which are exactly identical to the preceding sample.

Finally, because our experiment was not set up for accurate measurement of temporal precision or latency we here do not report any *temporal measures* of data quality. For further reference, two recent studies specifically investigated latency in the Vive Pro Eye HMD and found gaze tracking delays of approximately 50 ms (54) and 58 ms (53), respectively.

### Analysis

We report accuracy and precision metrics after aggregation at multiple levels. First, we summarize the distribution of repeated samples, gaze angles, and spatial quality metrics across all individual trials (targets), independent of the experimental session or participant they were recorded from. This analysis gives a broad overview of aggregate eye tracking performance expected from a typical behavioral experiment across a large part of the headset’s FOV. Second, we then average metrics within each session (across all 74 presented target positions) and investigate how factors such as the specific HMD used and vision correction worn by each participant influence eye tracking performance within a session, including with regard to target eccentricity. Before aggregating at this level, we removed individual trials with an absolute angular error of 5° or greater as outliers, motivated by the fact that our targets were spaced at a minimum distance of 5°. We additionally removed all trials in which one or both eyes had a large fraction ( > 90%) of repeated data samples (see below). Finally, we describe how each person’s inter-pupillary distance (IPD) can be estimated using the recorded eye position data.

To analyze the general effect of vision correction and compare HMDs with regard to accuracy and precision, we used a 3 × 2 linear model with between-subjects factors *vision correction* (glasses, contacts, or no correction) and *HMD* (1 or 2). For vision correction, we tested the hypothesis that contact lenses or glasses should yield lower spatial accuracy and precision than uncorrected vision, whereas for the factor HMD the null hypothesis assumed no performance differences between two units of the same hardware. To further illustrate the spatial pattern of data quality across the field of view, we split the data into the 6 between-subjects groups described above and plot the spatial distribution of accuracy and precision across the FOV. All statistical analyses were based on an alpha level of 0.05, and the Holm method was used to correct for multiple comparisons whenever necessary.

All data as well as Python and R code for the experiment and analysis are available at https://osf.io/gahcp/ (52).

## Results

### Gaze Sample Validity

**Figure 2.**
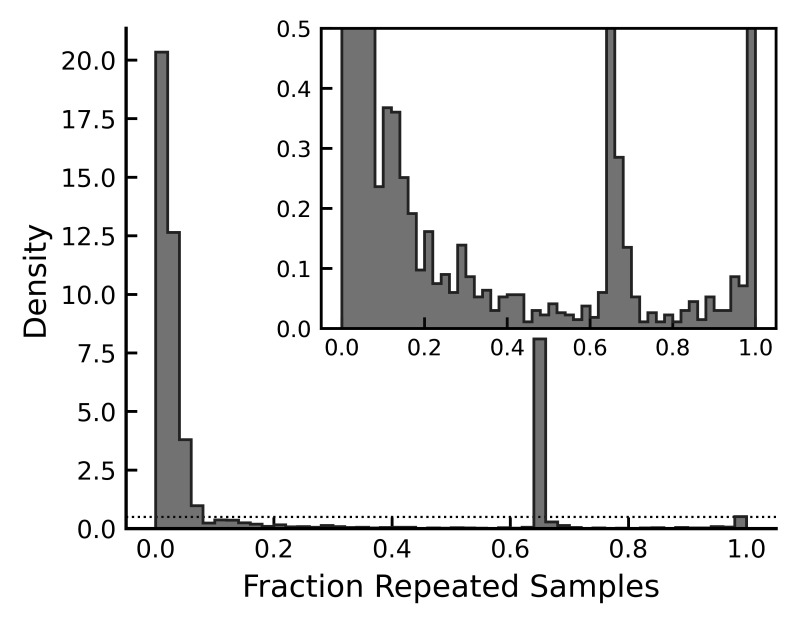
Frequency density histograms of the fraction of repeated samples in each trial (an indirect measure of invalid samples). The inset in the top right corner displays the same histogram with the y axis zoomed to a maximum value of 0.5 (dotted line) to better visualize small frequency densities beyond the three major clusters. Data shown includes all trials regardless of whether the left, right, or combined gaze representation had repeated samples.

As described above, we first computed the rate of identically repeated gaze samples in each trial as a proxy for sample validity (cf. 22). Figure 2 shows a frequency density histogram of the rate of repeated samples across all trials (regardless of whether data from the left eye, right eye, or combined gaze samples was repeated), highlighting three main clusters of data. Most trials (10479 trials, 79%) showed fewer than 20% of repeated samples. Although this measure cannot distinguish between individual reasons for sample repetition, this is a reasonable range of values to assume causes such as eye blinks, individual data recording errors, or spurious repeated values. In a small number of trials (131 trials, 0.9%), 100% of the samples recorded from one eye were identical while the other eye had a much lower rate of repetition, suggesting that one eye was closed or could not be correctly tracked by the eye tracker’s camera. Because missing data from one eye could bias the combined gaze representation, we decided to exclude these trials from further analysis. Finally, 2280 trials (17%) fell into a range between 60% and 70% sample repetition (mean: 65.0%), visible as a peak in the histogram (Figure 2). When analyzed further, gaze traces from these trials showed intermittent sample repetition ("stuttering") instead of large chunks of repeated data. However, spatial distributions and eye movement traces of these trials were comparable to trials not affected by this phenomenon, and results of statistical analyses remained qualitatively similar if these trials were temporarily excluded. Therefore, we did not remove these trials from final analysis and discuss possible explanations in more detail in the Discussion section.

### Raw Fixation Data and Outlier Correction

All participants were able to successfully complete the eye tracking calibration procedure in every session. As a first characterization of overall eye tracking performance, we computed summary statistics for accuracy and precision (measured as both SD and RMS) pooled across all recorded fixation trials, independent of which participant they were recorded from (74 targets × 10 sessions × 18 participants = 13320 trials). These metrics were computed before any correction for outliers and missing monocular data took place (see below). Individual trial accuracy values thus ranged from 0.09° to 28.04° (mean: 1.22°; median: 0.86°; interquartile range (IQR): 0.55° - 1.41°). SD precision ranged from 0.03° to 29.13° (mean: 0.43°; median: 0.21°; IQR: 0.14° - 0.34°), and RMS precision from 0.014° to 14.47° (mean: 0.23°; median: 0.08°; IQR: 0.05° - 0.15°).

As mentioned above, we then removed any trials with fixations showing an absolute angular error of 5° or larger as outliers (237 trials, 1.8%), and trials in which one or both eyes had a large fraction ( > 90%) of repeated samples as invalid data (131 trials or 0.9%). In total, 368 trials (2.76%) were removed from further analysis due to these criteria. All results reported below are based on the outlier-corrected dataset. Out of the trials removed due to gaze errors larger than 5°, the majority came from participants who wore glasses (205 of 237 trials, 86%). Visual inspection of the outlier trials’ gaze sample data revealed many instances of highly variable or erratic data, suggesting that occlusion or distortion by the glasses’ lenses or rims might play a role in data loss. Other outlier trials showed that participants looked at the target location very late in the trial or not at all. Finally, for some trials at the most far peripheral target locations, participants’ gaze was stable but deviated too far toward the periphery, suggesting that at least some of these instances might be caused by peripheral HMD lens distortion.

### Overall Accuracy and Precision

**Figure 3.**
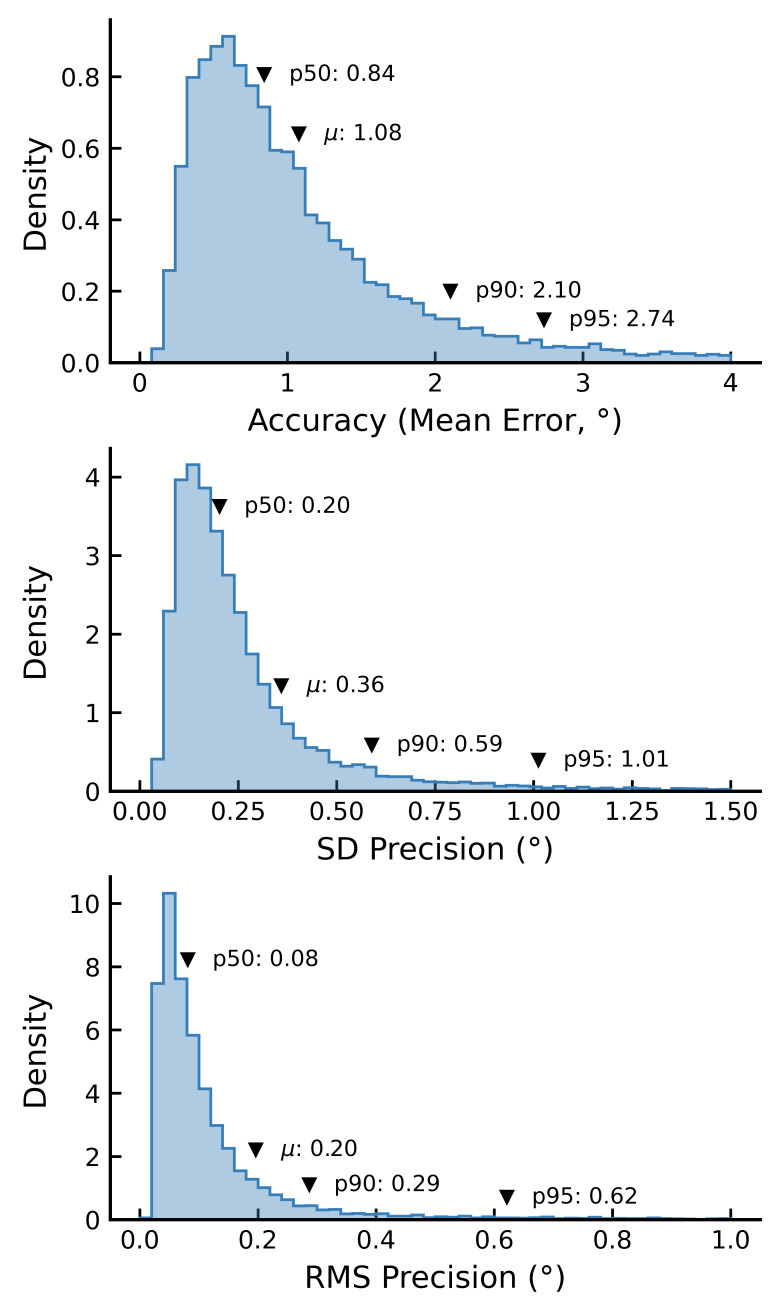
Frequency density histograms of accuracy and precision metrics across all validation trials (combined gaze). Top: Accuracy (average absolute error relative to target), Middle, Bottom: Precision (standard deviation and inter-sample root mean square error of gaze angles). Histograms are based on fixations to all presented target positions. Means and percentiles for each measure are annotated to allow direct comparison with device specifications. Data computed before outlier correction. Note: Plots are truncated at a specific x value (4°, 1.5°, and 1°, respectively) to better visualize the overall distributions and percentiles.

Final gaze accuracy after correcting for outliers lay between 0.09° and 4.99° (mean: 1.08°; median: 0.84°; IQR: 0.54° - 1.35°) across all fixations from all participants. SD precision lay between 0.03° and 8.98° (mean: 0.36°; median: 0.20°; IQR: 0.13° - 0.32°), and RMS precision was between 0.014° and 8.85° (mean: 0.20°; median: 0.08°; IQR: 0.05° - 0.14°). Figure 3 displays the resulting frequency density histograms for all three measures based on the combined gaze representation as reported by the SRanipal SDK, together with their mean and 50th, 90th, and 95th percentiles.

The Vive Pro Eye’s technical specifications report a spatial accuracy of 0.5° - 1.1° "within FOV 20°" (25). Our mean accuracy of 1.08° after outlier correction falls within this specified accuracy range (Figure 3). For a more direct comparison, we also computed the same metrics after selecting only the target positions that fell within the inner 20° of FOV (i.e., removing targets at positions 15° from center). With this “inner target set”, we found a mean accuracy of 0.97° after outlier correction. While it is unclear whether the manufacturer’s numbers refer to standard deviations around the mean, quantiles, or absolute range, the interquartile range in our outlier-corrected data came relatively close to the reported values (full targets: 0.54° - 1.35°; inner targets: 0.50° - 1.20°).

When computed separately for each eye, gaze error and variability were generally larger than for the combined gaze data but comparable between eyes in each metric (monocular data not shown in Figure 3). Corrected for binocular outliers, accuracy in the left eye ranged from 0.10° to 24.01° (mean: 1.59°; median: 1.11°; IQR: 0.68° - 1.90°) compared to 0.12° - 45.35° in the right eye (mean: 1.45°; median: 1.02°; IQR: 0.66° - 1.61°). A similar pattern was found for SD precision (left: 0.04° - 25.89°, mean: 0.51°; median: 0.24°; IQR: 0.16° - 0.40°; right: 0.04° - 21.40°, mean: 0.49°; median: 0.22°; IQR: 0.15° - 0.36°) and RMS precision (left: 0.02° - 13.92°, mean: 0.28°; median: 0.09°; IQR: 0.06° - 0.17°; right: 0.02° - 21.67°, mean: 0.28°; median: 0.09°; IQR: 0.06° - 0.15°). Note that outlier correction was performed only on the binocular (combined) gaze data. Since the process used by the SRanipal SDK to combine data from both eyes is undocumented, large values for monocular accuracy and precision likely reflect trials on which the system was able to compensate for unreliable data in one eye using data from the other eye.

**Figure 4.**
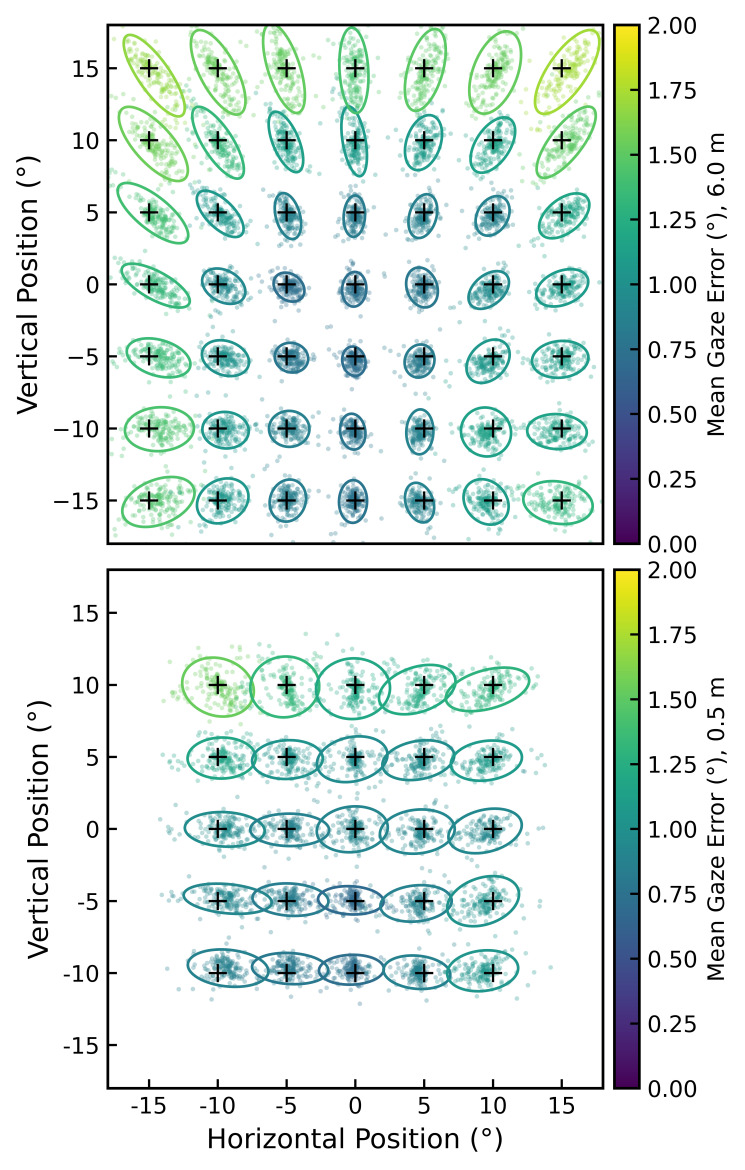
Average gaze position and 95% confidence ellipses for each target position, shown separately for the 6 m (top) and 0.5 m depth plane (bottom). Color scale illustrates average gaze accuracy in degrees across all trials presenting the corresponding target. Black crosses indicate target positions (cross arms approximate 1°).

To investigate the overall spatial pattern of gaze errors relative to each target position across the field of view, Figure 4 plots all 74 target angles relative to the HMD (split across depth planes; top: 6 m, bottom: 0.5 m viewing distance) together with the average gaze angle recorded in each trial (yielding 180 values per target, minus removed outliers). Additionally, Figure 4 includes 95% (± 2 standard deviations) confidence ellipses for each target. Ellipses and fixations are color-coded depending on each target’s average accuracy. In the far depth plane (top plot), gaze error increased from an average of 0.71° just below the central position (0°/-5°) to 1.72° in the upper right corner (+15°/+15°). Average errors were generally larger towards the upper compared to the lower periphery, and confidence ellipses indicate a radial error pattern with the major axes oriented outward from the central position. In the near depth plane (lower plot), average error was more equally distributed over the FOV and ranged from 0.69° (at 0°/-5°) to a maximum of 1.54° at the top left (-15°/+15°). Here, ellipse major axes indicate the greatest variability along the horizontal axis. Average SD precision in the far depth plane was also smallest near the center (0.24° at -5°/0°) and generally highest along the top row of targets (maximum: 0.51° at -15°/15°), with a similar distribution in the near depth plane (minimum: 0.28° at -5°/0°, maximum: 0.44° at -10°/10°).

### Data Quality for Participants and Sessions

**Figure 5.**
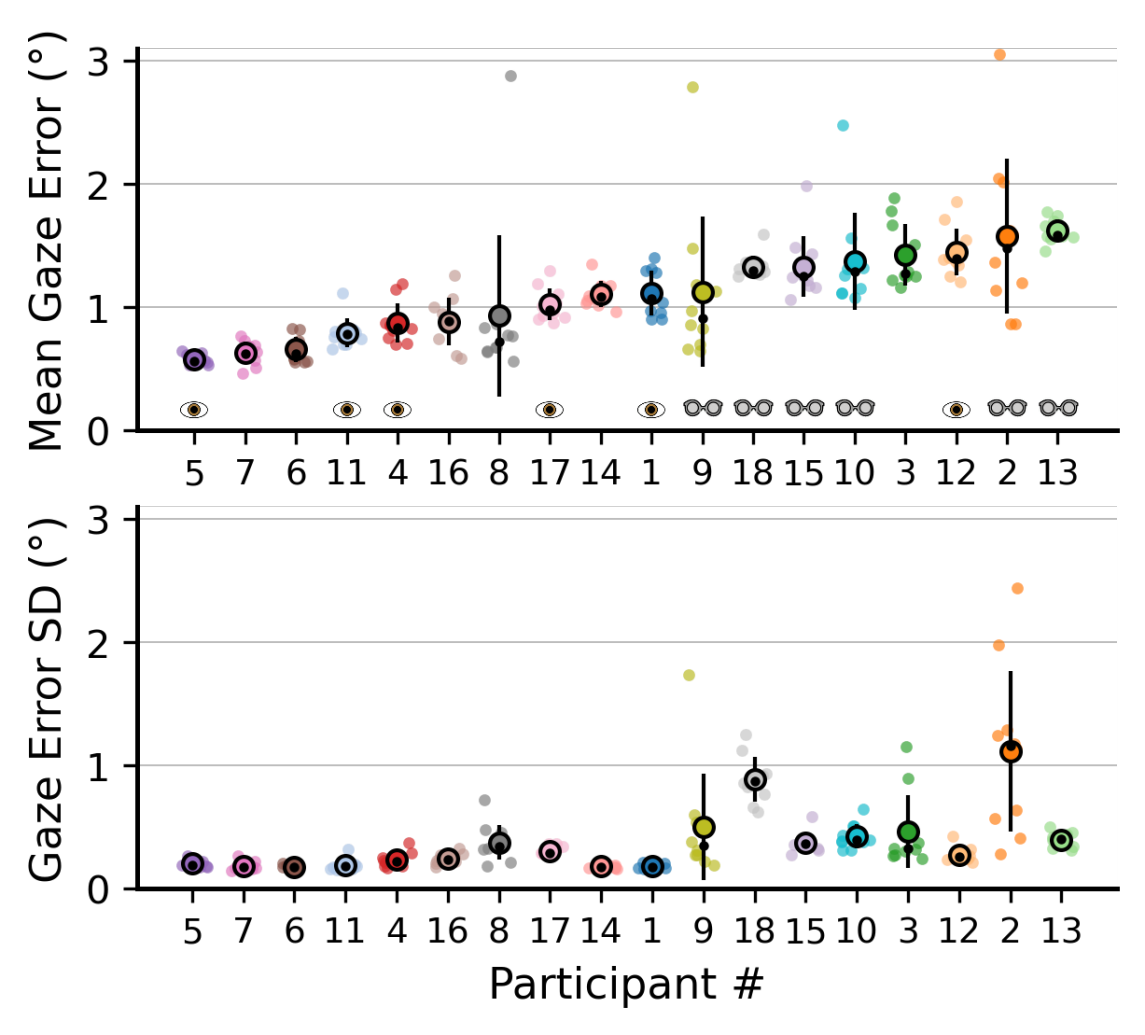
Accuracy (mean gaze error, top) and precision (standard deviation, bottom) of all individual participants. Small colored circles represent individual validation sessions. Large, open circles and error bars indicate mean and standard deviation across sessions, small black circles the median. Participants are sorted by average accuracy, numbers follow Table S1. Icons in the top panel indicate that a participant wore glasses or contact lenses (eye icon). Icons designed by OpenMoji (openmoji.org) under CC-BY-SA 4.0 license.

As a next step, we aggregated data quality metrics over all presented fixation targets within each measurement session of each participant. If the previous analysis on the level of individual targets approximates an experiment with multiple participants and sessions, each aggregated dataset here more closely represents the performance achievable by a given participant in an experimental session, at least as long as visual stimuli are generally presented within the tested target range. The resulting metrics are shown in Figure 5 for accuracy (top panel) and SD precision (bottom panel). Small markers here indicate values from individual sessions, while large markers and error bars indicate participant means and standard deviations across all ten sessions, thus serving as a measure of individual calibration reliability across repeated testing. Participants are sorted by their individual average accuracy, which ranged from 0.58° to 1.62° (SDs: 0.18°-1.12°). Icons below individual data indicate whether this participant wore contact lenses (eye icon) or glasses (glasses icon) during their measurement sessions.

Participants showed very consistent average accuracies across sessions, suggesting that they are likely to reach comparable eye tracking performance when calibrated and tested multiple times, for example over the course of multiple study sessions. Individual accuracy ranges (defined as the difference between the "best" and "worst" session accuracy of a given participant) ranged from 0.12° to 2.32°. Most participants’ accuracy range spread fell below 1°, with the exception of four persons who showed very noticeable outliers in one of their sessions (participants 2 and 8-10; Figure 5, top). If these "outlier sessions" were excluded, calibration ranges for all participants would be within 0.92°. Angular gaze precision (SD) in each session (cf. Figure 5, bottom) ranged from 0.14° to 2.44°. With decreasing accuracy, precision was also reduced: Gaze SD values increased with higher mean gaze error, and both measures were moderately correlated across participants (r = .62, p = .006, R² = 0.379).

### Vision Correction and HMD Hardware

**Figure 6.**
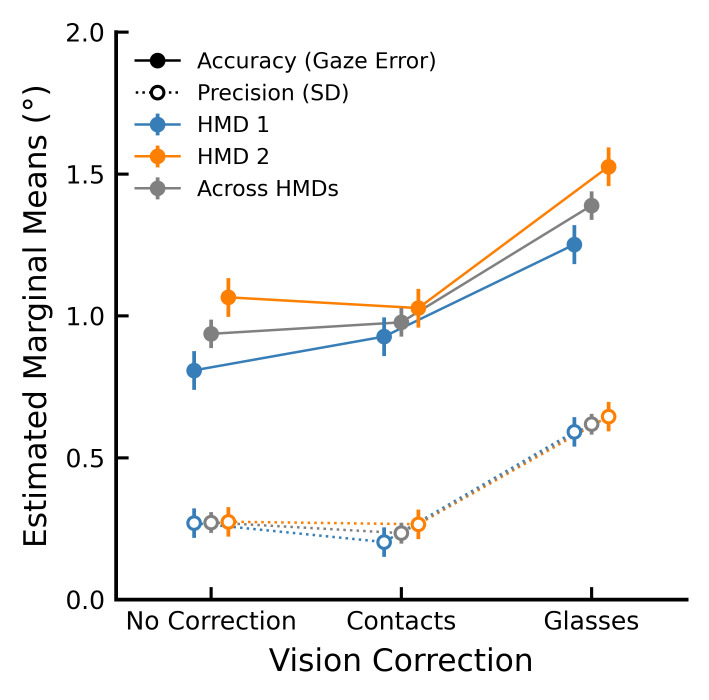
Estimated marginal means resulting from linear models on aggregated session data, each comparing the effects of vision correction and HMD. Model results are shown for accuracy (mean gaze error; filled markers) and precision (SD; open markers). Error bars indicate ±1 SEM.

Figure 5 suggests that participants who wore glasses had lower gaze accuracy compared to those with contacts or uncorrected vision, evidenced by higher average gaze errors. To more closely evaluate how vision correction influences accuracy and precision metrics and explore whether individual HMD units differ in eye tracking performance, we fit a linear model with the factors vision correction × HMD. Estimated marginal means from this analysis for both accuracy and precision are shown in Figure 6 (colored markers and lines).

For average gaze error as a measure of accuracy (filled markers and solid lines in Figure 6), the model indicated a significant main effect of vision correction (F_2,174_ = 26.8, p < .001, η²_p_ = .235). Gaze errors were significantly larger for glasses than for either contacts (t_174_ = -6.02, p < .001) or no vision correction (t_174_ = -6.61, p < .001), while contacts and no vision correction were not significantly different (t_174_= -0.59, p = 0.554). Accuracy also differed between individual HMDs, with HMD 1 showing on average 0.21° lower gaze error (F_1,174_ = 14.3, p < .001, η²_p_ = .076). There was no interaction between vision correction and HMD (F_2,174_ = 1.0, p = 0.376) for spatial accuracy.

With regard to precision (gaze error SD; open markers and dotted lines in Figure 6), vision correction again showed a significant effect (F_2,174_ = 33.2, p < .001, η²_p_ = .276). Similar to accuracy, glasses were associated with higher variability (lower precision) than either contact lenses (t_174_ = -7.39, p < .001) or uncorrected vision (t_174_= -6.67, p < .001), with contacts and no vision correction not significantly different (t_174_ = 0.73, p = .469). HMDs did not differ significantly in their measured precision (no main effect; F_1,174_ = 0.9, p = 0.340), and vision correction and HMD also showed no evidence of an interaction (F_2,174_ = 0.2, p = 0.834).

The two tested HMD units showed a small but significant difference in accuracy, but not precision. As discussed below, this might reflect an actual difference in hardware or, more likely, be related to individual participants in the sample despite our efforts to balance vision correction and device. Under the assumption that the effect is likely to be sample-related, we also report results of a reduced linear model for accuracy and precision which only included the factor vision correction (collapsing data across HMDs; cf. Figure 6, gray markers and lines). For accuracy, this model also yielded a significant main effect of vision correction (F_2,177_ = 24.9, p < .001, η²_p_ = .220). Glasses still had significantly larger gaze errors than contact lenses (t_177_ = -5.80, p < .001) and no vision correction (t_177_ = 6.38, p < .001), while contacts and uncorrected vision remained similar (t_177_ = 0.57, p = 0.568). When we compared both models directly using a standard F-test, the full model (vision correction × HMD) for accuracy fit the data significantly better than the reduced model (F_3,174_ = 5.4, p = 0.0014; adjusted R²_full_ = .266; adjusted R²_reduced_ = .211). Results of the reduced model for SD precision were also comparable to the full model, showing a main effect of vision correction (F_2,177_ = 33.5, p < .001, η²_p_ = .275) and the same pattern between correction types in post-hoc analysis (glasses – contacts: t_177_ = 7.43, p < .001; glasses – no correction: t_177_ = 6.70, p < .001; contacts – no correction: t_177_ = -0.73, p = 0.467). For SD precision, adding the factor HMD did not lead to a significant improvement in model fit over the reduced model (F_3,174_ = 0.4, p = 0.735; adjusted R²_full_ = .259; adjusted R²_reduced_ = .267).

**Figure 7.**
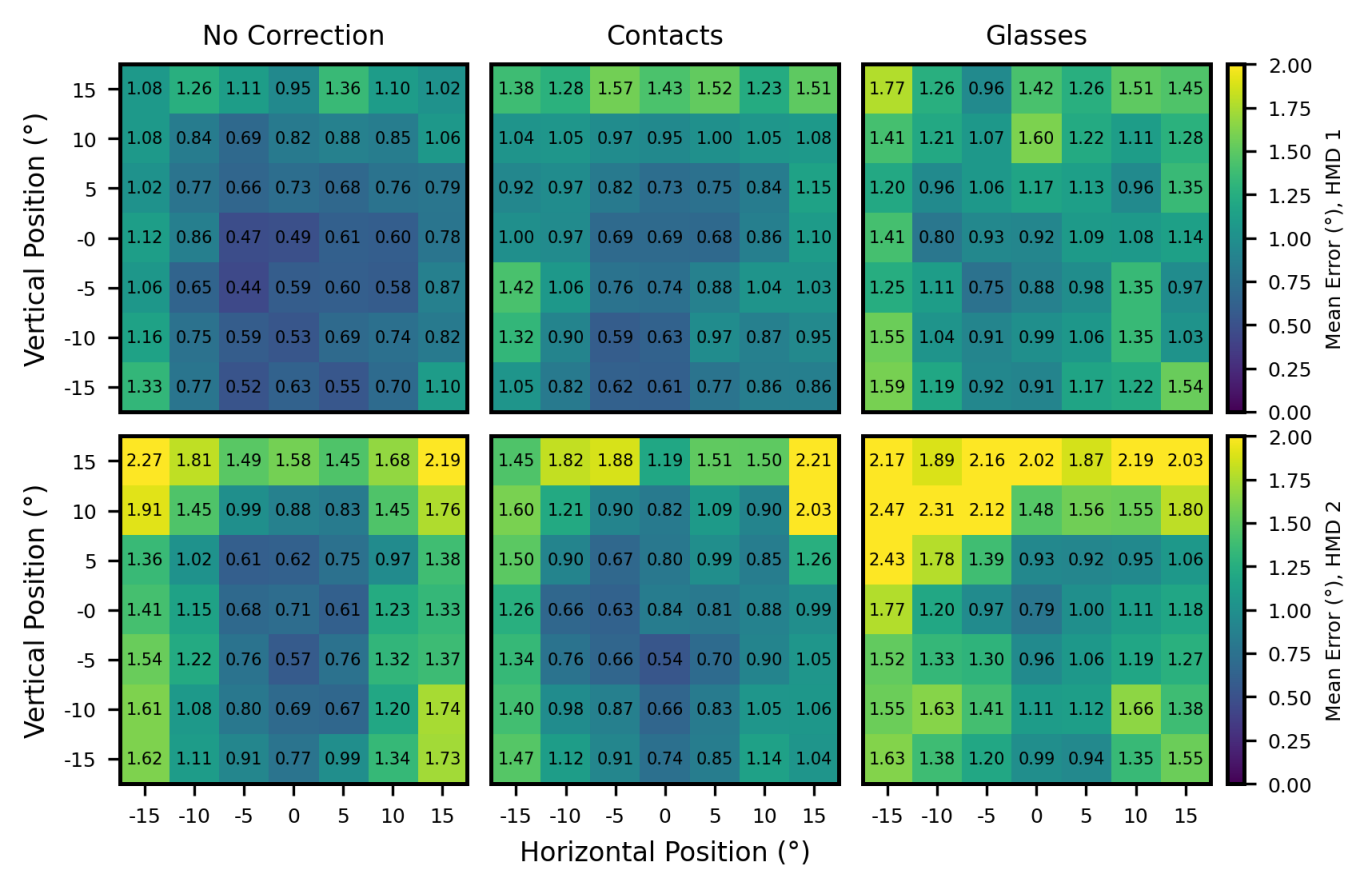
Average gaze accuracy at each presented target position across the FOV, split between HMD 1 (top row) and HMD 2 (bottom row), as well as by vision correction (columns)

Finally, because the linear model analysis found significant main effects of vision correction and HMD on gaze accuracy, we further split the spatial accuracy data shown in Figure 4 by the same two factors. The resulting matrix plots (Figure 7) illustrate the spatial distribution of gaze accuracy over the visual field for each HMD and vision correction group. Glasses show larger error along the periphery independent of the HMD used, and HMD2 shows generally larger errors with a strong tendency to reduced accuracy along the upper rows of targets.

### Inter-Pupillary Distance

**Figure 8.**
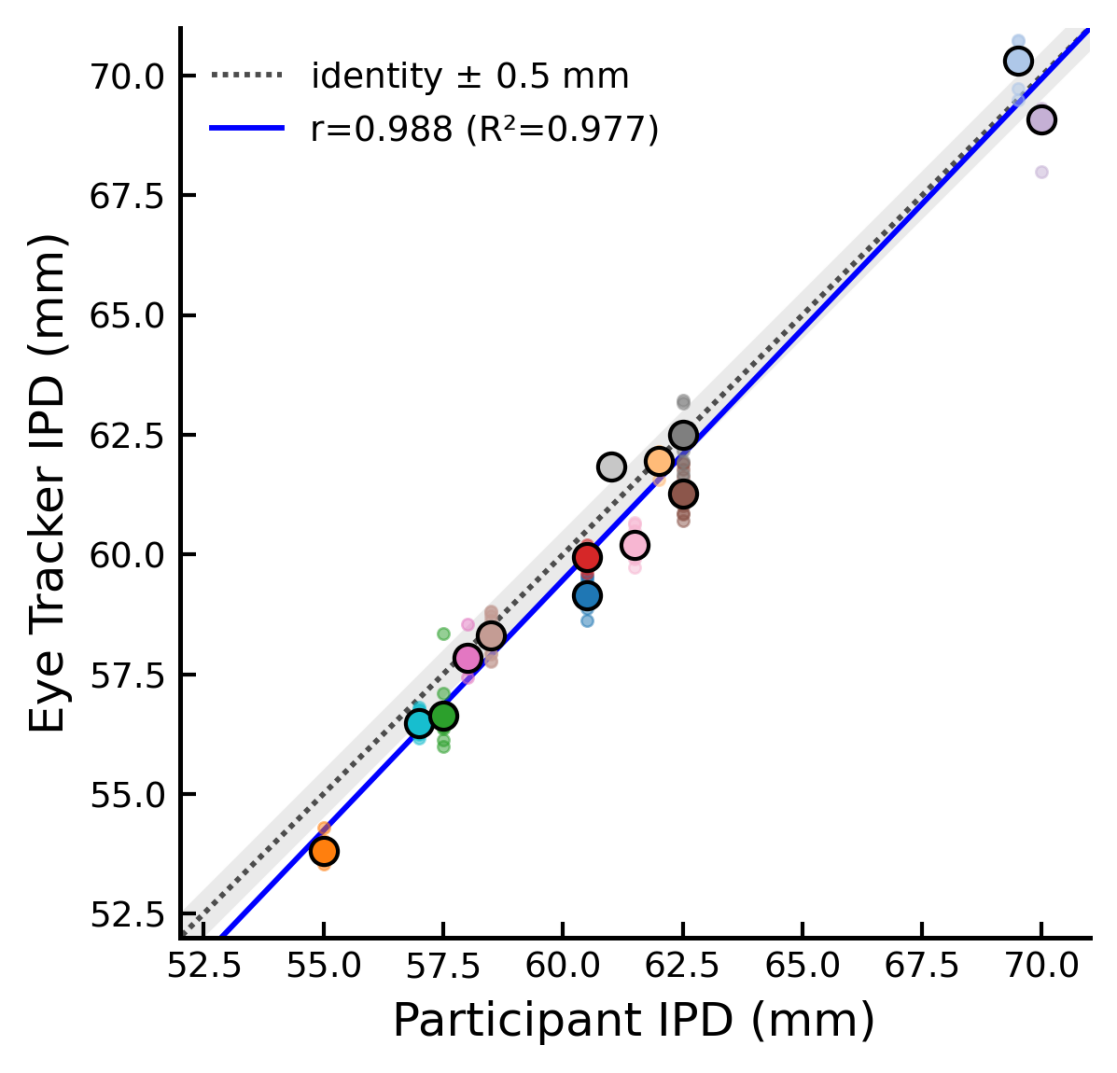
Participants’ inter-pupillary distance (IPD) estimated using eye tracker data, plotted as a function of true optometric IPD measured using a pupilometer. Small markers represent separate measurement sessions, large markers indicate average estimated IPD (used for regression analysis). Note that optometric data was only available for fourteen participants, as it was recorded after the study was concluded.

We extracted average 3D positions of the left and right pupil in the eye tracker’s frame of reference for each session and computed an estimate of IPD as the mean horizontal distance between both pupil positions. Figure 8 shows the individual sessions and mean IPD values extracted from gaze data as a function of each participant’s actual IPD value when measured using a pupilometer.

Actual participant IPDs ranged from 55 mm to 70 mm (mean: 61.1 mm; median: 60.8 mm), which is in good agreement with data previously estimated from a larger population (9). However, the range of physical IPD settings on the Vive Pro Eye is approximately 60 mm to 72 mm, meaning that five participants fell outside of the range supported by the HMD’s lenses. Average participant IPDs measured using gaze data ranged from 53.8 mm to 70.3 mm (mean: 60.7 mm; median: 60.1 mm) and were highly correlated with participant’s optometric IPD values (r = .988, p < .001, R² = .977; cf. Figure 8).

Gaze-based IPD values were on average smaller than optometric measures, with a mean error of -0.48 mm (standard deviation: 0.70 mm; range -1.35 mm - 0.83 mm).

## Discussion

Here, we present an in-depth evaluation of the real-world eye tracking performance achieved using the HTC Vive Pro Eye’s built-in eye tracking system. Overall, we found the built-in calibration to be highly reliable as evidenced by the highly reproducible post-calibration accuracy for most participants (Figure 5). The device’s spatial accuracy was found to be in accordance with the official device specifications. All participants were successfully calibrated, independent of vision correction, but accuracy and precision were significantly reduced for participants wearing glasses. Additionally, there was a small but significant difference in accuracy between the two tested devices. Gaze accuracy and precision were generally highest near the center of the field of view and decreased towards the periphery, and this pattern was further influenced by the choice of vision correction.

When analyzing individual fixations independent of participant and session, we found an average binocular accuracy of 1.08° for the Vive Pro Eye. Additionally, the average binocular gaze accuracy across participants in this task was 1.10°, with some individual participants’ mean gaze errors as low as 0.58°. While not as accurate as tower-mounted systems operated by a skilled experimenter, this is a good level of performance, especially since a consumer eye tracking device is likely to be focused on robustness across a large range of users rather than optimized for accuracy (23; 39). The human oculomotor system explores visual scenes by moving the fovea, the region of highest visual acuity which spans approximately 1-2° of visual angle, from one location of interest to the next (18). Based on this approximation of foveal size, 0.5° of spatial error is often given as a good threshold accuracy in eye tracking studies in order to be able to reliably identify the current fixation location (22, 23). In some of our participants, individual average accuracy was quite close to this rule of thumb, especially when not wearing glasses. However, the present level of accuracy currently precludes the analysis of small fixational eye movements such as drifts and microsaccades (36; 46).

Researchers should therefore carefully control the size of scene objects within a complex virtual environment (8), or adapt the size of gaze target areas in a gaze interaction or selection task. As an example, the minimal target size to achieve 80% capture rate as defined by Orquin and Holmqvist (41) is predicted by their heuristic as 3.7° - 10.1° for our range of participants (7.1° if computed using the across-participant average accuracy of 1.1° and SD precision of 0.37°). Similarly, the model in Schuetz et al. (51) predicts a minimal target size of approximately 3.5° for the same capture rate, also using across-participant average metrics (note that both papers acknowledge that the models use simplified assumptions and may be specific to circular stimuli and/or a specific eye tracker model). These and similar predictions for minimal stimulus size need to be taken into account when designing an interaction task for the Vive Pro Eye.

As mentioned above, participants’ average gaze accuracy was at the upper end of the published specifications (0.5° - 1.1°; 25), yet the exact circumstances under which these values were measured are not mentioned anywhere. In relation to published academic accounts, the average accuracy measured here was comparable to other values found for this hardware (1; 49), with the exception of the work by Sipatchin et al. (53) who report much larger average gaze errors of 4.16° for head-fixed target presentation. This difference might be related to the larger range of target eccentricities they presented (up to 26.6°) due to their goal of approximating a visual perimetry task. At the same time, both Sipatchin et al. (53) and our data show a clear increase in gaze error with increasing target eccentricity, and their values for 13° of eccentricity are comparable to our 15° targets in the present study. These findings further underline the importance of reporting multiple data quality metrics including a distribution of error across the FOV (17; 23), as a single measure of "average accuracy" does not adequately capture an eye tracking system’s performance in all situations. Another possible explanation for differences in average accuracy between the two studies could lie in the software used: Sipatchin and colleagues (53) report using the Tobii Pro SDK, which allows access to unfiltered gaze data but is not available within the Vizard VR engine, while our experiment was based on HTC’s SRanipal SDK. If the latter applied significant filtering by default, this could naturally bias our results toward greater accuracy while introducing other effects such as added latency that would not have been detectable in our experimental paradigm. Sipatchin et al. (53) did not find evidence of temporal filtering, but future work might still compare the spatial metrics achieved using both SDKs in a more direct manner. Finally, drifts or eye blinks during the longer fixation interval used in their paradigm (4.5 s of analyzed gaze data, compared to 1 s in our study) might also explain some of the difference in accuracy. In any case, the fact that the first few device evaluation studies for VR eye tracking hardware yield such diverging results with different approaches merely underlines the need for further work in this area.

A general pattern of decreasing gaze accuracy for peripheral compared to central targets as shown in Figure 4 has previously been reported in screen-based eye tracking systems (17; 23; 24; 39). This suggests that a peripheral decrease in accuracy is not specific to HMD-based devices. At the same time, the small form factor of an HMD could necessitate more oblique views of the eye tracking camera onto the pupil and increase subsequent gaze misestimations (known as pupil foreshortening; 12). Additionally, residual distortions from the optical lens system have been shown to influence perception even after appropriate correction (56) and likely also affect eye tracking calibration. Figure 4 also reveals interesting qualitative differences between targets presented in different depth planes. For the far depth plane, where gaze directions were almost parallel due to targets being shown at optical infinity, the radial error pattern seen in Figure 4 is comparable to what has been reported before (17; 23; 24; 39; 51; 53). However, when targets were located in the near plane (at 0.5 m distance and therefore at a much steeper vergence angle), confidence ellipses were instead dominated by variability along the horizontal axis. The actual field of view of the eye tracking cameras inside the HMD is unknown, but for eyes verging this close the pupils might end up near the nasal edge of the range visible to the camera, again causing pupil foreshortening and distortion effects and leading to a noisier estimate of pupil position and gaze direction. Alternatively, since we report the combined binocular estimate in Figure 4, this horizontal variability might also reflect noisier integration of monocular data into the combined ("cyclopean") gaze vector. In any case, gaze targets in a VR study should be presented at sufficient distance to achieve a more stable estimate, with targets used for calibration ideally at optical infinity (6 m or 20 ft.). Future VR devices might also support distance-dependent calibration, especially once variable-focus display technology becomes generally available for VR and AR (e.g., 27).

As suggested by prior work (23; 39), we found reduced accuracy and precision when participants wore vision correction compared to when they did not. However, only glasses caused a statistically significant increase in gaze errors and variability, whereas participants with contact lenses were comparable to those with no vision correction on all metrics despite previous reports of reduced accuracy with contact lenses (39). It is possible that effects of contact lenses become detectable only when using a well-calibrated tower-mount eye tracking system with its correspondingly high spatial accuracy. For practical applications, we could not see a disadvantage to measuring participants with contact lenses, whether numerically or anecdotally based on calibration success. The significant impact of glasses could be explained by a number of additional optical factors, such as further image distortions and reflections from the additional lens layer between eye and camera or physical impedance of the eye tracking camera’s view by glasses frames. During testing, two anecdotal observations helped significantly improve calibration success and metrics for glasses-wearing participants: First, glasses had a tendency to fold the light blocking rubber flaps at the bottom of the headset against the HMD’s lenses, blocking eye tracking illuminators and in some cases part of the participant’s visual field. In these cases, folding the flaps down while donning the headset generally allowed successful calibration and improved eye tracking performance. Second, both HMD lenses and glasses had a tendency to fog up especially during colder temperatures, likely exacerbated by the requirement to wear surgical masks. This mostly happened on the first session of a measurement day (cf. also the "outlier sessions" in Figure 5), and letting the HMD "warm up" for some time reduced fogging issues. Following these provisions, all participants with glasses could successfully complete calibration in our study (albeit at the reported lower levels of accuracy and precision).

Interestingly, we found a significant difference between individual headsets when it came to accuracy (but not SD precision), with HMD 1 being more accurate than HMD 2 by 0.2° on average. This is still likely to be a spurious effect, dependent on the assignment of participants to HMDs or individual headset fit issues, as the number of individual participants tested with each HMD was still relatively low (9 per HMD). Nevertheless, future studies should compare metrics recorded on each HMD in a within-subjects design with a consistent group of participants to rule out true differences in gaze estimation accuracy. In any case, we recommend a quick data quality evaluation similar to the metrics measured here for a newly bought HMD or before a large-scale eye tracking experiment, to guard against differences in accuracy but also against hardware defects.

With all eye tracking methods, it is generally impossible to know where a participant is truly fixating at any given moment. Therefore, a potential limitation of our paradigm (and others like it) is that the assumption of accurate fixation on the presented calibration or validation target has to be treated as “ground truth” in absence of any external reference measurement, and any error or variability herein are reflected in the measured accuracy and precision. Future work could evaluate the Vive Pro Eye hardware using other approaches such as one based on smooth pursuit eye movements (cf., 5; 19). Pursuit eye movements require a moving target stimulus, thus allowing to more easily validate during the experiment that the participant is correctly following the target.

Taken together, we have found the Vive Pro Eye a capable eye tracking device for behavioral experimentation in virtual environments, as long as care is taken to adjust stimulus and task properties to the achievable eye tracking performance. We hope that the metrics summarized above can serve as a starting point for study design and encourage other researchers and manufacturers to publish similarly detailed metrics in the future.

### Ethics and Conflict of Interest

The author(s) declare(s) that the contents of the article are in agreement with the ethics described in http://biblio.unibe.ch/portale/elibrary/BOP/jemr/ethics.html and that there is no conflict of interest regarding the publication of this paper.

### Acknowledgements

This work was supported by the German Research Foundation (DFG) grant FI 1567/6-1 TAO ("The active observer"), “The Adaptive Mind”, funded by the Excellence Program of the Hessian Ministry of Higher Education, Research, Science and the Arts (both awarded to KF) and the DFG Collaborative Research Centre SFB/TRR 135 (Grant Number 222641018).

## supplementary material


