# Supplementary material for: Eye Tracking in Virtual Reality: Vive Pro Eye Spatial Accuracy, Precision, and Calibration Reliability
Source: J Eye Mov Res. 2022 Sep 7;15(3):10.16910/jemr.15.3.3. doi: 10.16910/jemr.15.3.3 (PMC10136368; doi:10.16910/jemr.15.3.3)
Supplement: Supplementary file 1 [file jemr-15-03-c-SD1-01.pdf]

Supplementary Materials

Table S1. Participant Demographics, Vision Correction, and Across-Session Eye Tracking Performance

Participant								Prescription		Accuracy		SD Prec.		RMS Prec.	
#	Gender	Age	HMD	Lab	Days	Vision	IPD	Left	Right	M	SD	M	SD	M	SD
1	M	36	1	3	7	C	60.5	-3.50	-3.00	1.12	0.19	0.18	0.02	0.09	0.02
2	F	22	2	2	5	G	55.0	-1.75	-2.75	1.58	0.67	1.12	0.69	0.75	0.47
3	F	30	2	1	5	-	57.5	-	-	1.43	0.26	0.46	0.31	0.27	0.22
4	F	26	1	1	3	C	60.5	-2.50	-2.50	0.87	0.17	0.24	0.06	0.11	0.05
5	M	31	2	1	2	C	-[a]	-0.50	-0.50	0.58	0.04	0.20	0.03	0.08	0.03
6	M	28	2	2	4	-	62.5	-	-	0.66	0.11	0.18	0.02	0.08	0.01
7	F	21	1	3	3	-	58.0	-	-	0.63	0.10	0.19	0.04	0.08	0.02
8	M	38	1	3	3	-	62.5	-	-	0.93	0.69	0.38	0.15	0.22	0.10
9	M	32	1	1	2	G	-[a]	-2.50	-2.50	1.13	0.64	0.50	0.46	0.30	0.37
10	F	20	2	3	3	G	57.0	-1.50	-2.50	1.38	0.41	0.43	0.10	0.22	0.06
11	F	33	1	3	3	C	69.5	-3.25	-2.75	0.79	0.13	0.19	0.05	0.07	0.03
12	M	30	2	3	1	C	62.0	-1.75	-1.75	1.45	0.20	0.27	0.06	0.14	0.05
13	M	22	2	2	1	G	-[a]	-0.50	-0.50	1.62	0.10	0.39	0.06	0.15	0.02
14	F	23	2	2	2	-	-[a]	-	-	1.11	0.11	0.18	0.02	0.07	0.01
15	M	31	1	1	2	G	70.0	-2.0	-2.0	1.33	0.26	0.37	0.08	0.17	0.05
16	F	25	1	2	3	-	58.5	-	-	0.88	0.21	0.24	0.04	0.11	0.03
17	M	27	2	1	2	C	61.5	-4.0	-6.0	1.02	0.14	0.31	0.04	0.13	0.02
18	F	49	1	2	1	G[b]	61.0	-0.5	-0.5	1.33	0.10	0.88	0.19	0.73	0.18
M		29.1					61.1			1.10	0.25	0.37	0.13	0.21	0.10
SD		7.2					4.3			0.33	0.21	0.25	0.18	0.21	0.13

Note. Participant accuracy and precision (in degrees) are computed as mean (M) and standard deviation (SD) across all ten measurement sessions per participant. Data based on all 74 presented target locations. Age given in years; HMD – head mounted display used; Lab – lab hardware used with this participant (cf. Methods); Days – number of individual testing days; IPD – inter-pupillary distance at optical infinity (in mm); Prescriptions given in diopters; M – male; F – female; C- contact lenses; G - glasses.

[a] Not measured in this participant.
[b] This participant wore bifocals, prescription values given for the far viewing part of the lenses.
